# Combination of inspiratory and expiratory muscle training in same respiratory cycle versus different cycles in COPD patients: a randomized trial

**DOI:** 10.1186/s12931-018-0917-6

**Published:** 2018-11-20

**Authors:** Wenhui Xu, Rui Li, Lili Guan, Kai Wang, Yuhe Hu, Limei Xu, Luqian Zhou, Rongchang Chen, Xin Chen

**Affiliations:** 1 Department of Respiratory Medicine, Zhujiang Hosptial, Southern Medical University, 253 Gongye Road, Guangzhou, 510282 China; 2 Department of Respiratory Medicine, The State Key Laboratory of Respiratory Disease, National Clinical Research Center for Respiratory Disease, Guangzhou Institute of Respiratory Health, First Affiliated Hospital of Guangzhou Medical University, 151 Yanjiang Road, Guangzhou, 510120 China

**Keywords:** Chronic obstructive pulmonary disease, Inspiratory muscle training, Expiratory muscle training, Combined respiratory muscle training

## Abstract

**Background:**

Difference between combined inspiratory and expiratory muscle training in same respiratory cycle or different cycles remained unclarified. We explored the difference between both patterns of combined trainings in patients with COPD.

**Methods:**

In this randomized, open-label, controlled trial, stable COPD subjects trained for 48 minutes daily, for 8 weeks, using a monitoring device for quality control. Ninety-two subjects were randomly and equally assigned for sham training, inspiratory muscle training(IMT), combined inspiratory and expiratory muscle training in same cycle(CTSC) or combined inspiratory and expiratory muscle training in different cycles(CTDC). Respiratory muscle strength, as the primary endpoint, was measured before and after training. Registry: ClinicalTrials.gov (identifier: NCT02326181).

**Results:**

Respiratory muscle training improved maximal inspiratory pressure(PImax), while no significant difference was found in PImax among IMT, CTSC and CTDC. Maximal expiratory pressure(PEmax) in CTSC and CTDC was greater than IMT(*P* = 0.026, and *P*=0.04, respectively) and sham training (*P* = 0.001). IMT, CTSC, and CTDC shortened inhalation and prolonged exhalation(*P* < 0.01). Subjects with respiratory muscle weakness in IMT and CTDC exhibited greater increase in PImax than those without. IMT, CTSC and CTDC showed no difference in symptoms and quality of life scales among themselves(*P* > 0.05).

**Conclusion:**

Both patterns of CTSC and CTDC improved inspiratory and expiratory muscle strength, while IMT alone only raised PImax. Respiratory muscle training might change the respiratory cycles, and be more beneficial for COPD patients with inspiratory muscle weakness.

## Introduction

Patients with COPD generally suffer from respiratory muscle dysfunction [1]. Severe respiratory muscle dysfunction can lead to problems such as dyspnea, hypoxemia, and decreased exercise capacity. Respiratory muscle dysfunction is closely related to the mortality of patients with COPD [2, 3]. Therefore IMT has been suggested as an important solution to decreased respiratory muscle function [4]. It has shown that IMT in patients with COPD can delay deterioration of lung function via increasing inspiratory muscle strength and endurance, which relieves dyspnea, and improves quality of life [5, 6].

However, in addition to impaired inspiratory muscle function, expiratory muscle fatigue may also occur in COPD. Increasing intrathoracic pressure and diminishing lung volume, expiratory muscle contraction promotes effective cough [7], which has been previously thought to be associated with airway clearance [8]. It is known that expiratory muscles are usually activated at the end of expiration in COPD patients during rest, or weight-bearing breathing [9]. And this helps to maintain respiratory function [10]. Although at present there is a few of researches examining expiratory muscle training(EMT), EMT alone or the combination of EMT and IMT was recommended to strengthen inspiratory and expiratory muscles [11].

Nowadays, few studies are focusing on combined inspiratory and expiratory muscle training in COPD patients, and the effect of combined training remains unclarified. Combined respiratory muscle training has been categorized into two patterns: CTSC(training both inspiratory and expiratory muscle in same respiratory cycle) and CTDC(training inspiratory and expiratory muscle separately in different respiratory cycles). Weiner P [12] allocated COPD patients to CTDC for 3 months and found it had no additional benefit compared to IMT. In contrast, Battaglia E [13] connected the target flow inspiratory muscle trainer with the expiratory muscle trainer for CTSC, and found it significantly improved respiratory muscle function in patients with COPD. Presently, it is unclear whether CTSC and CTDC provide additional rehabilitation benefits compared with IMT alone, and whether there are differences between the two patterns of combined respiratory muscle training. Thus, the study was to explore the rehabilitation effects of CTSC, CTDC, and IMT alone, using a modified threshold respiratory muscle trainer with monitoring device which allows inspiratory and expiratory muscle training in same respiratory cycle. As IMT, but not EMT, is the recommended method for respiratory muscle training at present, EMT group wasn't established in this trial.

## Materials and methods

### Study design

The trial was carried out at Zhujiang Hospital of Southern Medical University from January 2015 to December 2017. The protocol was approved by the Zhujiang Hospital ethics committee(number: 2016-HXNK-005), and consisted with the Declaration of Helsinki. Informed consents were signed and obtained from all the subjects before the trial. Prior to the trial, it was registered on ClinicalTrials.gov (identifier: NCT02326181). Report of this trial was consistent with the Consolidated Standard of Reporting Trials statement [14].

### Recruitment of subjects

During a run-in period for 2 weeks, Patients with clinically stable COPD [15], naive to pulmonary rehabilitation and willing to participate were eligible. Patients got excluded if they had cognitive disorders, organ failure, malignant tumors, or metabolic diseases.

**Fig. 1:**
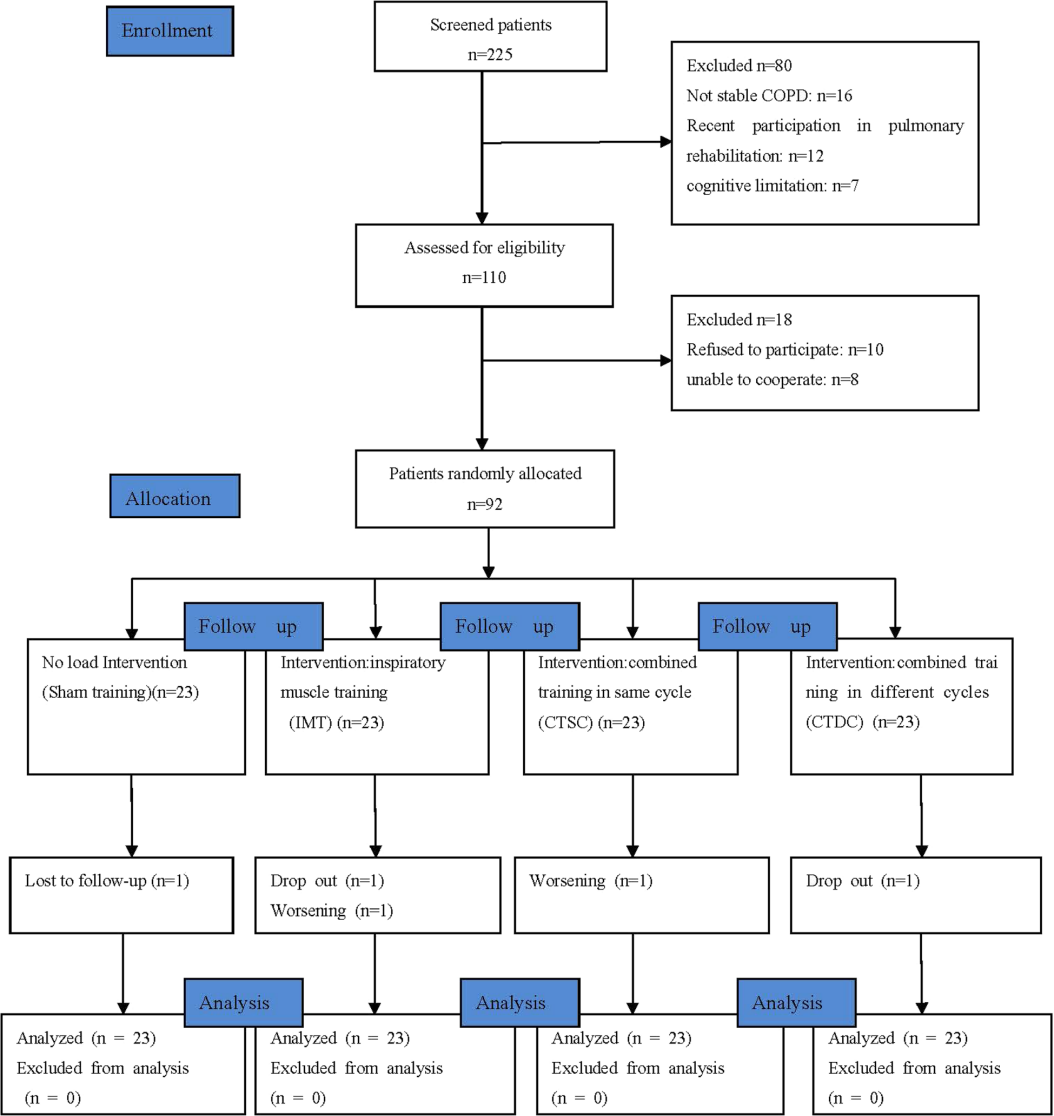
Flow diagram of the study

Of the 225 COPD subjects initially enrolled, 92 subjects were recruited and randomly allocated to Sham training, IMT, CTSC, and CTDC, according to a computer-generated sequence using a simple randomization method (Fig. 1). The randomization list was concealed in sequentially numbered, sealed, opaque envelopes and prepared by an independent physician not involved in subject recruitment. Each new subject was assigned a number sequentially, then the corresponding envelope was opened to decided which group they would enter. Before allocation, subjects learned all kinds of training. Subjects continued their regular medications during the study, and had the right to withdraw at any time.

### Study interventions

Subjects in all groups trained daily, with each session lasting for 48 minutes per day, 7 days a week, 8 weeks. The training was performed mainly at home, each set consisting of 3 minutes of training and 2 minutes of rest. Sham training performed 16 sets of no-load respiratory muscle training daily. IMT performed 8 sets of inspiratory muscle training and 8 sets of no-load respiratory muscle training per day. CTSC performed 16 sets of combined training in one respiratory cycle daily. CTDC performed 8 sets of inspiratory muscle training and 8 sets of expiratory muscle training separately in different cycles daily.

**Fig. 2:**
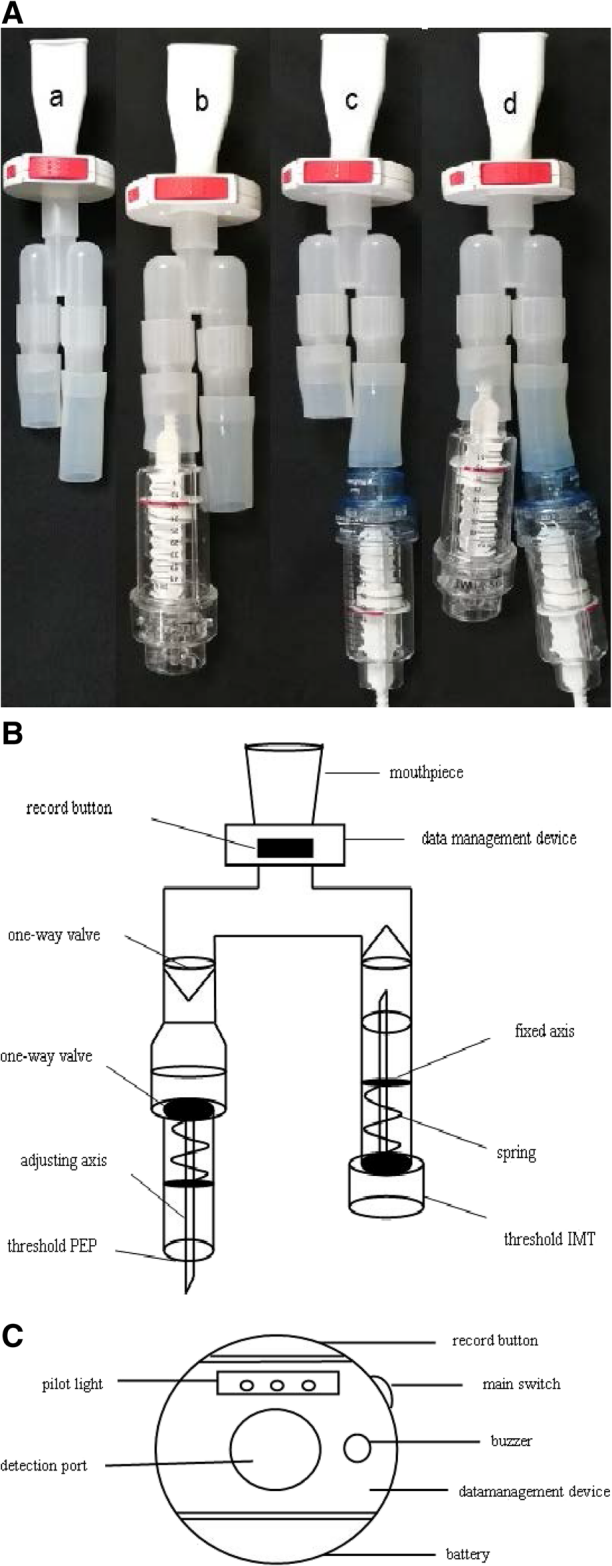
**A** Photo of the modified trainers./a, for no-load respiratory muscle training; b, for inspiratory muscle training only; c, for expiratory muscle training only; d, for concurrent respiratory muscle training. **B** General view of the modified trainer. /Threshold PEP and Threshold IMT(Respironics, Pittsburgh, USA) were respiratory muscle trainers designed for expiratory muscle training and inspiratory muscle training, respectively. **C** Coronal view of the data management device. /Main switch: powering up the data management device; Record button: recording training data when pressed; Pilot light: consisting of switch indicator(turning on when main switch is pressed) and time indicator(turning on when training is completed); Buzzer: ringing when training is nearly done; Detection port: detecting the airflow passing through; Battery: for energy supply

A modified threshold trainer with a monitoring device was used (Fig. 2-A). The modified trainer consisted of a threshold inspiratory trainer(Threshold IMT, Respironics, USA) and a threshold expiratory trainer(Threshold PEP, Respironics, USA), which were connected to a tube (Fig. 2-B) with two one-way valves(pattern ID:CN201721194926.4). Threshold trainers featured with an adjustable specific load [16]. Threshold PEP would be removed from the modified trainer to perform IMT. Threshold IMT would be removed to perform EMT. For sham training, both trainers would be removed. The load range of the modified trainer consisted with Threshold IMT(9-41 cmH_2_O) and Threshold PEP(5-20 cmH_2_O).

For training management, a monitoring device(pattern ID:CN201620070450.2) was installed (Fig. 2-C). The device recorded daily use including frequency and duration. Before training, subjects connected the device to a wireless terminal (such as a smart phone) through Bluetooth,which recorded daily use. The records were reviewed by staffs weekly. If a subject did not finish the planned assignment, they would take a lengthened training as compensation. Subjects were required to record, if any, discomfort during training. If their situation exacerbated, subjects would withdraw for treatment. Besides, Subjects received follow-up by telephone weekly and in the clinic every 2 weeks. In the clinic follow-up, Staffs checked the discomfort record, re-measure PImax and PEmax for load reset. The inspiratory load started at 30% PImax, and incrementally increased 5% every two weeks until reaching 45% PImax. The expiratory load was adjusted from 15% PEmax plus 5% PEmax every two weeks to 30% PEmax.

### Endpoints

The primary endpoint was respiratory muscle strength. The secondary endpoints were dyspnea, breathing pattern, spirometry, exercise capacity, quality of life, emotional status, BODE index, and nutritional status. Endpoints were measured before the start of intervention (as baseline) and within 7 days after its completion. All tests were performed by one same experienced respiratory physician blinded to the allocation.

### Respiratory muscle strength

Examination of respiratory muscle function consisted of measuring PImax and PEmax with a digital gauge (AZ-8205, AZ Instrument, Taiwan). Maximum value of three available tests that varied by less than 20% was recorded. The interval of each measurement lasted at 30 seconds at least. The lower limit of normal PImax was 60 cm H_2_O [17].

### Dyspnea

The modified Medical Research Council (mMRC) dyspnea scale was adopted to evaluate the severity of breathlessness.

### Breathing pattern

Breath flow rate was measured using a pneumotachograph (MLT300L, ADInstruments, Australia). Respiratory rate (RR), inspiratory time (Ti), expiratory time (Te), inspiratory time/total breath cycle duration (Ti/Ttot), tidal volume (Vt), and inspiratory capacity (IC) were acquired via calculating the flow rate.

### Spirometry

Spirometry was measured using a spirometer (PonyFX 229, Cosmed, Italy) which was calibrated daily according to the 2014 ATS guidelines [18]. Forced expiratory volume in one second (FEV_1_), percent-of-predicted FEV_1_(FEV_1_%), forced vital capacity (FVC), and FEV_1_/FVC were recorded before a bronchodilator test.

### Exercise capacity

Exercise capacity was assessed with 6-minute walk test (6MWT). During the test, heart rate and blood oxygen saturation were monitored by pulse oximetry. Oxygen saturation of all subjects was maintained at more than 90%.

### Quality of life, emotional status

Quality of life was reflected by St George’s Respiratory Questionnaire (SGRQ) and COPD Assessment Test (CAT) [19]; Hospital Anxiety and Depression Scale (HADS) were used for evaluation of emotional status.

### Nutritional status

Nutrition was reflected by BMI (body mass/height^2^) and FFMI (fat-free mass/height^2^). A FFMI ≤ 15 kg/m^2^ in women and FFMI ≤ 16kg/m^2^ in men was defined as low FFMI.

### BODE index

The BODE index consists of BMI (B), airflow obstruction (O), dyspnea (D), and exercise capacity (E), which was used for a comprehensive evaluation of the subjects.

### Statistical analysis

A value of *P* < 0.05 was considered statistically significant. For subjects who withdrew, an intention-to-treat analysis was done. The last observation carried forward method was used for data filling. Data were presented as mean ± standard deviation (SD) or mean ± standard error (SE) and analyzed via SPSS 20.0 (SPSS Inc., Chicago, USA). One-way analysis of variance (ANOVA) was applied for baseline comparison. A covariance analysis (ANCOVA) was adopted to analyze the differences (after minus before, shown as Δ) of each index, and models of least-significant difference were used for the baseline analysis. Differences in breathing patterns among groups and pre- and post-test comparisons were analyzed using mixed linear model. Subgroup analyses were performed using two-way ANOVA.

## Results

### Baseline characteristics

Ninety-two subjects initially participated in the study, but five dropped out. One subject in Sham training was lost to follow-up. Two subjects in IMT discontinued due to intolerance or deteriorated respiratory function, respectively. One in CTSC dropped out because of deteriorated respiratory function. One in CTDC was lost to follow-up. Basic data of all 92 subjects was not significantly different before the training (*P* > 0.05) (Table 1).

**Table 1:** Baseline demographic and clinical characteristics

	Sham training (*n*=23)	IMT (*n*=23)	CTSC (*n*=23)	CTDC (*n*=23)	*F*	*P-value*
Age(year)	69.43±6.44	67.49±6.17	68.26±7.03	67.22±7.35	0.498	0.685
Smoking index	513.04±559.47	356.52±389.44	380.87±448.49	355.22±446.89	0.603	0.614
BMI(kg/m^2^)	20.86±4.41	22.09±3.37	21.86±3.03	22.99±2.28	1.662	0.188
FFMI(kg/m^2^)	16.47±2.20	15.51±2.35	15.76±2.72	16.82±1.38	1.740	0.165
PImax>60cmH_2_O	72.73±12.59(n=12)	75.01±17.91(n=9)	75.79±9.79(n=8)	72.55±8.52(n=11)	0.211	0.888
PImax≤60cmH_2_O	46.12±11.03(n=11)	44.6±10.87(n=14)	43.83±10.41(n=15)	52.29±8.21(n=12)	1.795	0.161
PEmax (cmH_2_O)	63.82±18.46	60.26±19.96	56.10±16.73	62.13±13.56	0.844	0.473
mMRC	1.609±0.839	1.652±0.832	1.913±0.793	1.478±0.790	1.155	0.332
FVC(L)	2.312±0.781	2.229±0.673	2.076±0.813	2.298±0.593	0.517	0.671
FEV_1_(L)	1.137±0.462	1.161±0.325	0.967±0.292	1.120±0.339	1.375	0.256
FEV_1_%pred (%)	48.217±15.030	46.652±13.506	42.565±12.033	47.248±12.404	0.806	0.494
FEV1/FVC	0.501±0.123	0.534±0.108	0.505±0.172	0.495±0.112	0.554	0.648
6MWD(m)	415.130±52.301	398.957±78.733	382.087±81.637	400.652±78.016	0.776	0.510
CAT	10.478±5.846	10.696±4.106	12.609±4.131	10.348±5.149	1.094	0.356
SGRQ	16.435±7.415	17.348±6.184	18.783±5.018	17.565±7.051	0.511	0.676
HADS	5.870±3.293	5.174±2.125	5.696±2.835	5.000±3.568	0.436	0.727
BODE	2.870±1.576	3.304±1.636	3.826±1.154	2.783±1.930	2.614	0.062

Data are presented as mean± SD unless otherwise indicated

*Abbreviations*: *IMT* inspiratory muscle training, *CTSC* combined training in same cycle, *CTDC* combined training in different cycles, *BMI* body mass index, *FFMI* fat-free mass index, *PImax* maximal inspiratory pressure, *PEmax* maximal expiratory pressure, *mMRC* modified Medical Research Council, *FVC* forced vital capacity, *FEV*_*1*_ forced expiratory volume in 1 second, *%pred* percent predicted, *6MWD* 6-minute walking distance, *CAT* COPD Assessment Test, *SGRQ* St George’s Respiratory Questionnaire, *HADS* Hospital Anxiety and Depression Scale, *BODE* body mass index, airflow obstruction, dyspnea, and exercise capacity index

### Respiratory muscle strength

As shown in Table 2, the ΔPImax of IMT, CTSC, and CTDC group was significantly greater than Sham training (*P* < 0.05), but was not different among IMT, CTSC, and CTDC. Improvement of PEmax in CTSC and CTDC group was larger than IMT group (*P* = 0.026, and *P* = 0.04, respectively), and Sham training (*P* = 0.001), but CTSC was not significantly different from CTDC. No significant difference was found in the improvement of PEmax between IMT and Sham trainig (*P* = 0.218).

**Table 2:** Effects of respiratory muscle training on the IMT, CTSC, CTDC, and sham training

	Sham training (*n*=23)	IMT (*n*=23)	CTSC (*n*=23)	CTDC (*n*=23)	*F*	*P-value*
Δ BMI (kg/m^2^)	-0.084±0.489	-0.456±0.727	-0.145±0.534	-0.254±0.611	1.540	0.210
Δ FFMI (kg/m^2^)	-0.182±0.400	-0.231±0.537	0.128±0.913	-0.070±0.664	1.371	0.257
ΔPImax(cmH_2_O)	2.150±4.30	9.830±6.857^a^	8.722±6.052^a^	8.130±6.548^a^	7.347	<0.001
ΔPEmax (cmH_2_O)	2.160±5.107	4.544±4.119	8.844±8.155^a,b^	8.313±6.635^a,b^	5.710	0.001
Δ mMRC	0.130±0.548	-0.304±0.559^a^	-0.261±0.449^a^	-0.217±0.422 ^a^	3.558	0.018
Δ FVC(L)	-0.013±0.098	-0.011±0.175	0.007±0.138	-0.059±0.158	0.706	0.551
Δ FEV_1_(L)	-0.005±0.081	-0.020±0.109	0.020±0.120	-0.030±0.086	0.561	0.642
ΔFEV_1_%pred (%)	-0.261±3.427	-0.826±4.549	-0.174±5.789	-2.030±5.358	0.688	0.562
Δ FEV1/FVC	-0.002±0.034	-0.001±0.034	0.014±0.049	0.002±0.017	0.940	0.425
Δ 6MWD(m)	8.478±20.784	24.174±18.364	19.087±26.787	19.739±12.941	2.175	0.097
Δ CAT	0.304±2.098	-2.304±2.787^a^	-1.435±1.903^a^	-1.739±1.484^a^	6.575	0.0005
Δ SGRQ	-0.174±2.103	-3.087±2.429^a^	-2.565±1.927^a^	-2.261±1.322^a^	9.124	<0.001
Δ HADS	-0.174±1.370	-0.609±1.559	-0.652±1.849	-0.130±1.714	0.692	0.559
Δ BODE	-0.174±0.778	-0.478±1.163	-0.348±1.434	-0.174±1.230	0.472	0.703

*Abbreviations: IMT* inspiratory muscle training, *CTSC* combined training in same cycle, *CTDC* combined training in different cycles, *BMI* body mass index, *FFMI* fat-free mass index, *PImax* maximal inspiratory pressure, *PEmax* maximal expiratory pressure, *mMRC* modified Medical Research Council, *FVC* forced vital capacity, *FEV*_*1*_ forced expiratory volume in 1 second, *%pred* percent predicted, *6MWD* 6-minute walking distance, *CAT* COPD Assessment Test, *SGRQ* St George’s Respiratory Questionnaire, *HADS* Hospital Anxiety and Depression Scale, *BODE* body mass index, airflow obstruction, dyspnea, and exercise capacity index

Data are presented as mean± SE unless otherwise indicated; Δ, difference (after minus before intervention); ^a^*P*<0.05vs control group; ^b^*P*<0.05vs IMT group

### Dyspnea

The ΔmMRC of IMT, CTSC, and CTDC group were significantly improved compared to Sham training(*P* < 0.05). However, no significant differences among groups was shown (*P* > 0.05) (Table 2).

### Breathing pattern

As shown by Table 3, no significant difference was found among groups. After training, there was no significant change of breathing pattern in Sham training. CTSC, CTDC, and IMT were characterized by a decrease of Ti and Ti/Ttot, and an increase of Te, but there were no significant differences in Vt and IC. In addition, respiratory rate in CTSC group significantly reduced.

**Table 3:** Breathing pattern before and after respiratory muscle training

	Sham training (*n*=23)	IMT (*n*=23)	CTSC (*n*=23)	CTDC (*n*=23)	*P*_2_
RR(breaths/min)					
Pre-test	20.3 (18.6 to 21.9)	20.3 (18.7 to 22)	20.7 (19 to 22.3)	20.0 (18.3 to 21.6)	
Post-test	20.1 (18.6 to 21.6)	20.1 (18.6 to 21.6)	18.7 (17.1 to 20.2)	19.8 (18.3 to 21.3)	*P*_2_=0.949
*P*_1_	0.584	0.275	<0.01	0.466	
Ti(S)					
Pre-test	1.489 (1.353 to 1.624)	1.481 (1.346 to 1.617)	1.435 (1.299 to 1.570)	1.501 (1.366 to 1.637)	
Post-test	1.486 (1.354 to 1.617)	1.435 (1.303 to 1.566)	1.388 (1.257 to 1.520)	1.457 (1.325 to 1.588)	*P*_2_=0.851
*P*_1_	0.691	<0.01	<0.01	<0.01	
Te(S)					
Pre-test	1.751 (1.521 to 1.980)	1.719 (1.489 to 1.948)	1.687 (1.457 to 1.916)	1.661 (1.431 to 1.890)	
Post-test	1.755 (1.527 to 1.984)	1.757 (1.528 to 1.985)	1.752 (1.524 to 1.981)	1.723 (1.495 to 1.952)	*P*_2_=0.989
*P*_1_	0.571	<0.01	<0.01	<0.01	
Ti/Ttot					
Pre-test	0.466 (0.434 to 0.497)	0.474 (0.442 to 0.506)	0.476 (0.444 to 0.508)	0.488 (0.456 to 0.520)	
Post-test	0.466 (0.435 to 0.496)	0.460 (0.430 to 0.490)	0.457 (0.427 to 0.488)	0.470 (0.439 to 0.500)	*P*_2_=0.922
*P*_1_	0.886	<0.01	<0.01	<0.01	
Vt(L)					
Pre-test	0.469 (0.447 to 0.491)	0.467 (0.444 to 0.489)	0.463 (0.441 to 0.485)	0.479 (0.457 to 0.501)	
Post-test	0.465 (0.444 to 0.485)	0.471 (0.450 to 0.492)	0.462 (0.441 to 0.482)	0.477 (0.457 to 0.498)	*P*_2_=0.762
*P*_1_	0.127	0.127	0.645	0.539	
IC(L)					
Pre-test	1.613 (1.461 to 1.766)	1.568 (1.416 to 1.720)	1.541 (1.389 to 1.693)	1.665 (1.513 to 1.818)	
Post-test	1.600 (1.456 to 1.744)	1.575 (1.431 to 1.720)	1.537 (1.393 to 1.682)	1.652 (1.508 to 1.796)	*P*_2_=0.909
*P*_1_	0.068	0.313	0.634	0.077	

Data are presented as adjusted mean differences (95% confidence interval of the differences) unless otherwise indicated; *P*_1_ results of mixed linear model comparison of baseline versus week 8 in each group (within-subject effects); *P*_2_ results of mixed linear model comparison among the four groups(between-group effects)

*Abbreviations: RR* respiratory rate, *Ti* inspiratory time, *Te* expiratory time, *Ti/Ttot* inspiratory time/ total breath cycle duration, *Vt* tidal volume, *IC* inspiratory capacity

### Spirometry and exercise capacity

No significant changes were observed in ΔFVC, ΔFEV_1_, ΔFEV_1_%pred, ΔFEV_1_/FVC, and Δ 6MWD among groups (*P* > 0.05) (Table 2).

### Quality of life, emotional status

ΔSGRQ and ΔCAT indicated changes in quality of life. The ΔSGRQ and ΔCAT of IMT, CTSC, and CTDC were notably lower than Sham training (*P* < 0.05), but no significant difference among groups was found (*P* > 0.05). In addition, there was no significant difference in depression and anxiety scores among groups (*P* = 0.559) (Table 2).

### BODE index and nutritional status

Compared with Sham training, no significant improvements was observed in BODE index, BMI, and FFMI in IMT, CTSC, and CTDC groups (*P* > 0.05) (Table 2).

### Subjects with vs. without respiratory muscle weakness

Each group was divided into two subgroups according to respiratory muscle strength, and then a subgroup analysis was performed. Respiratory muscle weakness was defined as PImax < 60 cm H_2_O. As reported in Table 4, subjects with respiratory muscle weakness in IMT group benefited more in PImax than those without (*P* = 0.009). CTDC exhibited a benefit for PImax and PEmax (*P* = 0.038 and *P* = 0.007). There were not differences before and after training in all endpoints of CTSC and sham training (*P* > 0.05), except ΔFEV_1_%pred in CTSC.

**Table 4:** Effects of respiratory muscle training on COPD patients with or without respiratory muscle weakness

Subgroups	Sham training	IMT	CTSC	CTDC	ANOVA (interaction)
Number	23	23	23	23	
PImax>60cmH_2_O	12	9	8	11	
PImax≤60cmH_2_O	11	14	15	12	
Δ BMI(kg/m^2^)					
PImax>60cmH_2_O	-0.101±0.394	-0.593±0.770	-0.056±0.541	-0.173±0.618	
PImax≤60cmH_2_O	-0.066±0.595	-0.367±0.713	-0.192±0.543	-0.328±0.622	*P*=0.702
*p*	0.867	0.479	0.574	0.554	
ΔFFMI(kg/m^2^)					
PImax>60cmH_2_O	-0.188±0.431	-0.232±0.583	0.053±0.335	0.131±0.814	
PImax≤60cmH_2_O	-0.176±0.385	-0.230±0.528	0.168±1.118	-0.254±0.450	*P*=0.605
*p*	0.941	0.993	0.780	0.170	
ΔPImax(cmH_2_O)					
PImax>60cmH_2_O	1.758±4.911	5.367±5.558	5.738±5.218	5.218±6.244	
PImax≤60cmH_2_O	2.576±3.710	12.700±6.155	10.313±6.011	10.800±5.835	*P*=0.254
*p*	0.659	0.009	0.084	0.038	
ΔPEmax(cmH_2_O)					
PImax>60cmH_2_O	1.808±5.852	4.700±3.482	8.275±11.023	4.600±6.340	
PImax≤60cmH_2_O	2.544±4.404	4.443±4.606	9.147±6.593	11.717±5.007	*P*=0.162
*p*	0.739	0.888	0.814	0.007	
Δ mMRC					
PImax>60cmH_2_O	0.167±0.577	-0.333±0.707	-0.250±0.463	-0.273±0.467	
PImax≤60cmH_2_O	0.091±0.539	-0.286±0.469	-0.267±0.458	-0.167±0.389	*P*=0.938
*p*	0.749	0.847	0.935	0.559	
Δ FVC(L)					
PImax>60cmH_2_O	-0.033±0.114	0.004±0.208	0.051±0.184	-0.059±0.210	
PImax≤60cmH_2_O	0.009±0.078	-0.021±0.159	-0.016±0.107	-0.059±0.100	*P*=0.661
*p*	0.312	0.745	0.277	0.999	
Δ FEV_1_(L)					
PImax>60cmH_2_O	-0.015±0.072	-0.053±0.134	0.095±0.123	-0.021±0.104	
PImax≤60cmH_2_O	0.006±0.092	0.001±0.089	-0.021±0.101	-0.039±0.068	*P*=0.033
*p*	0.405	0.749	0.097	0.687	
ΔFEV_1_%pred(%)					
PImax>60cmH_2_O	-0.500±2.876	-2.556±5.812	2.875±4.764	-1.246±6.620	
PImax≤60cmH_2_O	0.0±4.074	0.286±3.292	-1.800±5.759	-2.750±4.048	*P*=0.074
*p*	0.539	0.256	0.024	0.620	
Δ FEV_1_/FVC					
PImax>60cmH_2_O	-0.003±0.033	-0.014±0.030	0.040±0.051	0.005±0.020	
PImax≤60cmH_2_O	-0.002±0.037	0.007±0.035	-0.001±0.044	-0.001±0.014	*P*=0.037
*p*	0.981	0.148	0.058	0.451	
Δ 6MWD(m)					
PImax>60cmH_2_O	3.667±25.850	25.556±25.793	15.750±17.958	15.909±7.687	
PImax≤60cmH_2_O	13.727±12.539	23.286±12.615	20.867±30.928	23.250±15.910	*P*=0.775
*p*	0.255	0.780	0.673	0.180	
Δ CAT					
PImax>60cmH_2_O	0.583±2.193	-2.333±2.121	-1.125±2.532	-1.636±1.433	
PImax≤60cmH_2_O	0±2.049	-2.286±3.221	-1.600±1.549	-1.833±1.586	*P*=0.962
*p*	0.518	0.969	0.581	0.759	
Δ SGRQ					
PImax>60cmH_2_O	0.167±2.588	-3.333±1.732	-2.250±2.493	-2.182±1.537	
PImax≤60cmH_2_O	-0.546±1.440	-2.929±2.841	-2.733±1.624	-2.333±1.155	*P*=0.811
*p*	0.430	0.706	0.579	0.791	
Δ HADS					
PImax>60cmH_2_O	-0.250±1.138	-0.556±1.333	-1.000±1.927	-0.364±1.963	
PImax≤60cmH_2_O	-0.091±1.640	-0.643±1.737	-0.467±1.846	0.083±1.505	*P*=0.924
*p*	0.788	0.899	0.523	0.545	
Δ BODE					
PImax>60cmH_2_O	-0.167±0.937	-0.556±1.014	-0.125±0.991	-0.364±1.286	
PImax≤60cmH_2_O	-0.182±0.603	-0.429±1.284	-0.467±1.642	0.000±1.206	*P*=0.803
*p*	0.964	0.805	0.598	0.492	

Data are presented as mean± SE unless otherwise indicated; Δ, difference (after minus before intervention)

*Abbreviations: IMT* inspiratory muscle training, *CTSC* combined training in same cycle, *CTDC* combined training in different cycles, *BMI* body mass index, *FFMI* fat-free mass index, *PImax* maximal inspiratory pressure, *PEmax* maximal expiratory pressure, *mMRC* modified Medical Research Council, *FVC* forced vital capacity, *FEV*_*1*_ forced expiratory volume in 1 second, *%pred* percent predicted, *6MWD* 6-minute walking distance, *CAT* COPD Assessment Test, *SGRQ* St George’s Respiratory Questionnaire, *HADS* Hospital Anxiety and Depression Scale, *BODE* body mass index, airflow obstruction, dyspnea, and exercise capacity index

## Discussion

### Respiratory muscle strength

In this study, two patterns of combined respiratory muscle training, CTSC and CTDC, were compared. Both groups were not significantly different in almost all endpoints, except the significantly reduced breathing rate in CTSC. Similar to Battaglia E [13], our trial also found that combined respiratory muscle training could strengthen inspiratory and expiratory muscle. In Battaglia’s work, patients received home based training twice daily for 15 minutes, 7 days a week, for 12 months with a target flow respiratory muscle trainer. The increase in PImax and PEmax was not significant until 6 months after training. However, in our study, the effects were significant after 8 weeks in CTSC and CTDC. As a target flow trainer usually requires a specific inspiratory flow which depends on breathing pattern, the outcomes may fluctuate. Nevertheless, a threshold pressure respiratory muscle trainer, independent of the flow, can regulate the training intensity precisely [16], making it more useful. Watsford M [20] applied the Powerlung device to explore the effect of CTSC on the recovery of healthy people. They adjusted the training load in accordance with subjects’ endurance, and discovered that CTSC could significantly improve both PImax and PEmax. Training loads of at least 30% PImax were required to improve respiratory muscle strength [21]. But no standard of load exists for expiratory muscle training. In our study, we used 15% PEmax as an initial load [12] and then the load raised incrementally, which gradually increased endurance of subjects.

The improvement of PEmax in CTSC and CTDC were significantly better than IMT in our work. Similarly, Weiner P [12] found IMT alone could not enhance expiratory muscle strength. Increased expiratory load was assumed to induce a larger end-expiratory lung volume (EELV) [22]. Therefore, expiratory muscle training might worsen dynamic lung hyperinflation through increasing EELV in COPD patients. But another research stated that expiratory load was not associated with lung hyperinflation [23]. EELV, or even dynamic pulmonary hyperinflation can be reliably reflected via IC [24]. No significant change in IC occurred in each group, which meant EELV did not change significantly after training. The mechanism behind airflow limitation with expiratory muscle recruitment has been yet completely clarified. Contraction of expiratory muscle during exhalation might be a nonspecific response to increased respiratory stimulus [25]. Despite lung hyperinflation, Abdominal muscle recruitment during expiration preserves fiber length and force-generating ability of diaphragm muscle for the onset of inhalation [10]. Therefore, inspiratory muscle may get fatigue if expiratory muscle dysfunction occurs.

### Breathing pattern

Through recorded breathing pattern, it’s a pity that we didn’t find any difference in breathing patterns between groups. But the pattern in CTSC, CTDC, and IMT was characterized by a decrease of Ti and Ti/Ttot, and an increase of Te. Prolonged exhalation probably relieves dyspnea by decreasing dynamic pulmonary hyperinflation [26]. A reduction in Ti/Ttot might increase diaphragm blood flow [27], thus taking more oxygen to important inspiratory muscles in diaphragm. But no difference was observed in VT of each group after training, and this may result from increasing contraction velocity of inspiratory muscles to maintain VT [28]. Different from CTDC, respiratory frequency of CTSC was significantly lower after training, which may be due to differences in the physiology among the groups.

### Dyspnea

Neuromechanical dissociation was a popular theory to explain the dyspnea caused by respiratory muscle dysfunction [29]. Dyspnea-associated physical activity limitation is a common complaint in COPD patients with moderate to severe airflow obstruction. Avoidance of activity to relieve dyspnea leads to a sedentary lifestyle, which ultimately causes a decline in exercise capacity. Because of inspiratory muscle fatigue and inefficiencies, COPD patients might use a high proportion of PImax to inhale, which might contribute a greater sense of dyspnea [30]. Held HE [31] suggested increase of expiratory muscle strength is beneficial to relieve dyspnea and improve quality of life. Therefore, combined respiratory muscle training helps to relieve dyspnea. In the current study, degree of dyspnea in CTSC, CTDC, and IMT group decreased, and respiratory muscle strength of these groups increased by different degrees. This indicated that respiratory muscle training can relieve dyspnea in COPD patients via strengthening their respiratory muscle.

### Quality of life

Decreased quality of life is a predictor for mortality and rehospitalization in COPD [32]. Studies have shown that both inspiratory and expiratory muscle training can improve quality of life in patients with COPD [8, 33]. We found that the improvement of SGRQ and CAT in CTSC, CTDC, and IMT was significantly greater than Sham training, probably owing to larger reductions in dyspnea after respiratory muscle training. Though there was no differences among CTSC, CTDC, and IMT. A study showed that the correlations among SGRQ, CAT, and mMRC were strong [33]. Therefore, dyspnea relieving from respiratory muscle training was beneficial to the amelioration of SGRQ and CAT.

### Spirometry and exercise capacity

No significant benefits for spirometry were found from respiratory muscle training. This might be related to the incompletely reversible airway obstruction and emphysema. On the other hand, It is well known that lower limb muscle dysfunction is a leading cause of decreased exercise capacity [34]. Many researchers found IMT helpful in restoring the exercise capacity of patients with COPD, but the effect didn’t reach clinical significance [5, 35, 36]. We also observed no remarkable changes in exercise capacity. It was recommended that, to enhance exercise capacity, inspiratory muscle strength should increase at least by 30% from the baseline level [37], according to which it is rational to observe no significant improvement in exercise capacity in our work. Subjects with inspiratory muscles weakness in IMT group and CTDC group exhibited greater increases in PImax, suggesting that respiratory muscle training may be more beneficial for COPD patients with impaired inspiratory strength, similar to a prior meta-analysis [5]. But no change was noted in CTSC group. It may result from some unknown physiological mechanisms, or it just takes longer for the change to become evident.

### Limitation

One major limitation of this study was that it was relatively small size. Due to the limited size, subgroup analysis could not be done in each group based on the revised ABCD assessment tool in 2017 Global Initiative for COPD [1]. Despite these, to the best of our knowledge, this is the first randomized controlled trial to compare combined respiratory muscle training in same respiratory cycle and different cycles with monitoring device in COPD patients. Although the monitoring device could not record threshold load, it provided reliable quality control for the training. Monitoring device enabled us to make sure that subjects finished their training as possible, thus offering a stable rehabilitation. Although the sample size was not enough for subgroup analysis, more subjects were included in our study compared to previous researches. Besides, we also used new indicators on nutrition, quality of life and breathing pattern. Even though we could not find the better training between CTDC and CTSC, we nevertheless provided evidences about combined respiratory muscle training. The effects of combined training on patients with different inspiratory muscle strengths would assist healthcare providers in establishing individualized respiratory muscle training program.

## Conclusion

In this trial, we found that two patterns of combined trainings could strengthen both inspiratory and expiratory muscle, while IMT alone did not change PEmax remarkably. We also showed that respiratory muscle training might improve the breathing pattern of patients with COPD, and the effect was more remarkable in patients with inspiratory muscle weakness. As breathing frequency in CTSC group was reduced significantly, it is needed to explore physiological mechanisms among different trainings. This study suggest that patients may benefit more from both patterns of combined trainings than IMT alone.

## Abbreviations

COPD: Chronic obstructive pulmonary disease
IMT: Inspiratory muscle training
EMT: Expiratory muscle training
CTSC: Combined inspiratory and expiratory muscle training in same cycle
CTDC: Combined inspiratory and expiratory muscle training in different cycles
PImax: Maximal inspiratory pressure
PEmax: Maximal expiratory pressure
mMRC: modified medical research council
SGRQ: St George’s Respiratory Questionnaire
CAT: COPD assessment test
RR: Respiratory rate
Ti: Inspiratory time
Te: Expiratory time
Ti/Ttot: Inspiratory time/total breath cycle duration
Vt: Tidal volume
IC: Inspiratory capacity
FVC: Forced vital capacity
FEV_1_: Forced expiratory volume in one second
FEV_1_%: Percent-of-predicted FEV_1_
6MWT: 6-minute walk test
HADS: Hospital Anxiety and Depression Scale
BMI: Body mass index
FFMI: Fat free mass index
EELV: End-expiratory lung volume
